# An Ab Initio Metadynamics Study Reveals Multiple Mechanisms of Reactivity by a Primal Carbon Cluster Toward Hydrogen and Ammonia in Space

**DOI:** 10.3390/nano15141110

**Published:** 2025-07-17

**Authors:** Dobromir A. Kalchevski, Stefan K. Kolev, Dimitar V. Trifonov, Ivan G. Grozev, Hristiyan A. Aleksandrov, Valentin N. Popov, Teodor I. Milenov

**Affiliations:** 1 “Acad. E. Djakov” Institute of Electronics, Bulgarian Academy of Sciences, 72 Tsarigradsko Chaussee Blvd., 1784 Sofia, Bulgaria; dobromirak@ie.bas.bg (D.A.K.); skkolev@ie.bas.bg (S.K.K.); dtrifonov@ie.bas.bg (D.V.T.); igrozev@gmail.com (I.G.G.); 2 Faculty of Chemistry and Pharmacy, Sofia University “St. Kliment Ohridski”, 1 J. Bourchier Blvd., 1164 Sofia, Bulgaria; haa@chem.uni-sofia.bg; 3 Faculty of Physics, Sofia University “St. Kliment Ohridski”, 5 J. Bourchier Blvd., 1164 Sofia, Bulgaria; vpopov@phys.uni-sofia.bg

**Keywords:** DFTB2, carbon cluster, metadynamics, hydrogenation, nitrogenation, amination, space

## Abstract

We present a theoretical model of the hydrogenation and amination of a primal carbon cluster of the tangled polycyclic type. Hydrogen atoms were introduced via H_2_, while the nitrogen source was NH_3_. The initial chemical processes were modeled using Born–Oppenheimer Molecular Dynamics. Metadynamics was employed to accelerate the saturation. The reactions were characterized in terms of barriers, topology, and intricate changes in the electronic structure. All transition states were identified. Multiple mechanisms for each type of reaction were discovered. Occasional unbiased changes in the carbon skeleton, induced by the guided processes, were observed. The initial addition reactions had no barriers due to the instability and high reactivity of the carbon structure. The final product of barrierless hydrogen saturation was C_25_H_26_. This molecule included multiple isolated double bonds, a medium-sized conjugated π system, and no triple bonds. Ammonia additions resulted in quaternary ammonium groups and primary amino groups. In the subsequent amination, a barrier appeared in fewer steps than in repetitive hydrogenation. The final product of barrierless saturation with NH_3_ was C_25_H_2_(NH_3_)_2_NH_2_. Further amination was characterized by a forward free-energy barrier of an order of magnitude larger than the reverse reaction, and the product was found to be unstable.

## 1. Introduction

The chemical processes between primal carbon clusters and small inorganic molecules in space contribute to the overall molecular presence in interstellar space. Reactions with hydrogen- and nitrogen-containing species are of special interest because they may provide a promising route to pre-biological products. Moreover, nitrogen-doped carbon structures have attracted the attention of both theoretical and applied science [1,2,3,4].

Of the 256 molecules identified in space, 204 have been observed in the interstellar or circumstellar medium [5,6]. Almost all of them contain hydrogen, while most contain carbon, and over a third contain at least one nitrogen atom. The first and simplest organic molecules to be discovered in space are CH (methylidyne) and CH^+^ (methylidyne cation) [7,8]. Unlike mono-atomic gas, single-nucleus ions, and simple allotropic forms, they were the first chemical species to be detected in the interstellar medium. CN (cyano radical) was independently reported in 1940 [9] and 1941 [10], while CN^−^ (cyanide anion) was only recently discovered [11]. Detections of additional small molecules of the considered elements include HCN (hydrogen cyanide) [12], HNC (hydrogen isocyanide) [13,14], CCN (cyanomethylidyne) [15], C_3_N (cyanoethynyl radical) [16,17], HCNH^+^ (protonated hydrogen cyanide) [18], CNCN (isocyanogen) [19], HC_3_N (cyanoacetylene) [20], HCCNC (isocyanoacetylene) [21], HNCNH (carbodiimide) [22], CH_3_CN (methyl cyanide) [23], HC_3_NH^+^ (protonated cyanoacetylene) [24], C_5_N (cyanobutadiynyl radical) [25], NH_2_CH_2_CN (aminoacetonitrile) [26], HC_7_N (cyanotriacetylene) [27,28,29], and HC_9_N (cyanotetraacetylene) [30]. Ring systems offer the potential for greater complexity and closer alignment with biological functions. The family of compounds found in space includes C_6_H_6_ (benzene) [31] and C_6_H_5_CN (benzonitrile) [32]. Intriguing small to medium-sized cyclic molecules have recently been detected in TMC-1: C_3_H_2_ (cyclopropenylidene) [33], C_5_H_6_ (cyclopentadiene) [29], C_5_H_5_CN (cyanocyclopentadiene) [34], C_5_H_5_CCH (ethynyl cyclopentadiene) [35], and *o*-C_6_H_4_ (ortho-benzyne) [36]. Even more intriguing are the polycyclic species detected in TMC-1: C_9_H_8_ (indene) [29] and two isomers of C_11_H_7_N (1- and 2-cyanonaphthalene) [37].

The partial radical character in interstellar molecules can contribute to a reduction in activation energy in various reactions. Two recent articles revealed the initial steps in graphene nucleation during adsorption. Both demonstrate the possibility of mechanisms involving conjugated, radical, carbon-rich species [38,39].

We delved into the chemical possibilities arising from a primal cluster originating in a pure carbon environment reaching a hydrogen- or ammonia-rich area. Our focus was on side-chain functionalization. We employed Born–Oppenheimer Molecular Dynamics (BOMD) and Metadynamics to enable alternative mechanisms and parallel and conjunctive processes in the simulation of reagents, possible intermediates, and conditions. The reactions were characterized in terms of barriers and topology. Intricate changes in the electronic structure were analyzed. All transition states (TSs) were found. Multiple mechanisms for each type of reaction were discovered. Occasional spontaneous changes in the carbon skeleton, induced by the biased processes, were accounted for. The degree of saturation due to consecutive additions up to the appearance of a forward free energy (FE) barrier was observed for hydrogenation and amination.

**Figure 1:**
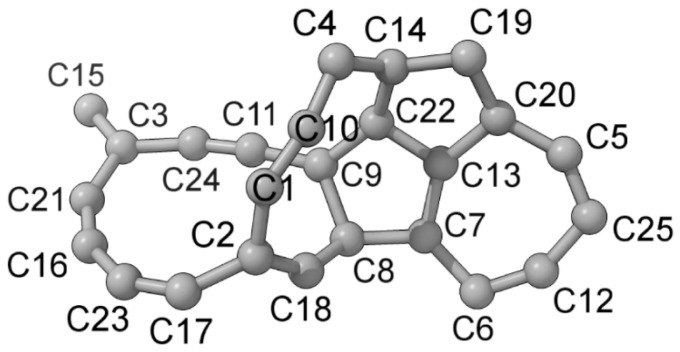
Atomic numbering of a C25 carbon molecule.

In this study, we report on the reactivity of a primal carbon cluster toward H_2_ and NH_3_ in space. The cluster results from the spontaneous relaxation of an aggregate of 25 randomly positioned C atoms. It is of a tangled polycyclic type. We generated it in a previous study [40] and successfully used it as a building block in the formation of closed-cage nanoparticles in the interstellar medium. The atomic numbering of C_25_ is given in Figure 1.

Amorphous carbon and hydrocarbon molecules have already been detected in space [41,42]. Although possessing a non-standard geometry, primal carbon clusters can still be easily classified as species of the tangled polycyclic type. To our knowledge, the reactivity of such molecules has never been experimentally or theoretically studied.

Our goal is to elucidate the properties and reactivity of carbon allotropes. Through theoretical chemistry, we aim to provide a thorough atomistic understanding of their chemical mechanisms at a level beyond that of current experimental possibilities. The instability of such carbon clusters is too high to allow for the isolation of products or intermediates. The conditions of the reactions prohibit an experimental examination of transition states. Some results are to clarify the general reactivity of carbon clusters, not only the reactivity toward H_2_ and NH_3_. We seek to contribute to the field of high-vacuum carbon chemistry by reaching conclusions that are sufficient to predict the most important aspects of behavior of primal carbon clusters. Our conclusions can be applied directly in laboratory setups or used indirectly as a stepping stone toward a better understanding of the chemistry of such systems.

## 2. Materials and Methods

In theoretical chemistry, atomic systems are viewed as structures composed of nuclei and electrons. Both components are considered to be particle waves, although quantum mechanics deals only with their wave properties. The only force taken into account is electromagnetic. Since a single electron occupies hundreds of thousands of times the volume of a nucleus, and the intensity of the electron wave is not evenly distributed in space, often the term used to describe this “cloud” of electrons is “electronic density”. For any given set of positions of the nuclei, there is an optimal distribution, energy-wise, of the electronic density. To discover it is the equivalent of finding the wavefunction of the molecule. The wavefunction can be used to calculate the value of every physical property of the system and, consequently, build the chemistry on top of the physics.

The wavefunction is computed in steps. One of the steps is to represent molecular orbitals (MOs) as a Linear Combination of Atomic Orbitals (LCAO). Another step is the Self-Consistent Field (SCF) method. The SCF method is an iterative procedure for orbital optimization. During this procedure, the coefficients of the atomic orbitals (represented as an LCAO) are systematically altered in order to minimize the energy of each MO. Once certain accuracy criteria (such as the energy change between two steps and a reduction in the energy in the last step) are met, the orbitals are considered optimized. In other words, an approximate solution to the wavefunction has been found for the given coordinates of the nuclei.

All calculations are performed with the CP2K/Quickstep 2023.1 software [43,44]. CP2K is a quantum chemistry program package, designed for atomistic simulations of gas-phase, liquid, and solid-state systems. It is capable of a wide palette of computations. These can range from predicting the physical properties of crystal materials and modeling organic reactions in explicit solvent models to enzymatic processes. Quickstep is a component of CP2K, designed for accurate and efficient DFT modeling of large, complex systems. It is employed in both static calculations and in ab initio molecular dynamics simulations.

The SCF optimizations are completed in the Self-Consistent Charge Density Functional-Based Tight Binding (SCC-DFTB/DFTB2) method [45]. An efficient a posteriori treatment for dispersion interactions is employed [46].

The DFTB method is an approximation of the Density Functional Theory (DFT), in which Kohn–Sham (KS) equations are transformed into tight-binding ones [47] related to the Harris functional [48]. The second-order expansion of the KS equations enables a transparent, parameter-free generalized Hamiltonian matrix. Its elements are modified by a self-consistent redistribution of Mulliken charges [45]. The KS energy additionally includes the Coulomb interaction between charge fluctuations. The accuracy of the DFTB method with self-consistent charge expansion is comparable to that of DFT methods and higher levels of the ab initio theory for various properties of single molecules, solutions, and solid-state materials, yielding satisfactory geometries and total energies [49,50,51]. DFTB produces realistic charge distributions, binding energies, and vibrational frequencies of charged solvated species [52]. The derived activation energies in organic chemistry also conform to higher levels of theory [52], enabling the study of reaction mechanisms. This method is found to yield excellent geometries and energetics for pure carbon species, such as fullerenes ranging from C_20_ to C_86_ [53], and, at the same time, is orders of magnitude less computationally intensive than DFT.

The simulations of the reactions are performed using BOMD [54] and Metadynamics (MTD) [55,56]. Metadynamics is a state-of-the-art simulation method in which the processes are guided to model a chosen chemical reaction. Collective variables (colvars; CVs) are defined over molecular degrees of freedom to bias the system toward selected changes. Penalty potentials (hills) are periodically generated for current values in the CV space to raise the free energy and to reach unexamined geometries. When a TS is crossed over, the study of the new minimum begins, starting again at the bottom of the energy pit. Reversing the bias potential peaks yields the relative stability of each geometry—the free-energy surface of the reaction. With the rise in energy, unguided changes can occur, providing realistic insights into the studied processes.

All the dynamics and metadynamics simulations are carried out in a canonical ensemble (NVT ensemble) with Periodic Boundary Conditions (PBCs). The thermostat uses canonical sampling through velocity rescaling (CSVR) [57,58], set for a temperature of 400 K. The timestep is 1 fs. The shortest intermolecular distances are above 3.4 Å in all the initial (zeroth) steps. Each simple dynamics run is preceded by cell and geometry optimization of the system. In MTD, the height of the Gaussian penalty potential is 1.255 kcal/mol. The scale factor (Gaussian width) for each collective variable is 0.2. Hills are generated every 50 fs. All walls are of the quadratic type with a potential constant of 20 kcal/mol. The temperature tolerance is always set to 50 K.

The TSs are located using dynamics methods, such as saddle points connecting the energy pits of reagents and products. In other words, the reaction coordinate corresponds to the energy maxima in every TS geometry. Their geometries correspond to the points in time, usually below 10 femtoseconds, when new bonds are formed and/or the old ones are dissociated, as presented in the figures below.

The initial dimensions of the explicit periodic cell are chosen on the basis of the size and shape of the studied primal carbon cluster, regardless of the type of reaction. The remaining vacant space of the PBC box are filled with either H_2_ or NH_3_ molecules. The final number of attacking molecules does not influence the mechanisms of the modeled reactions.

## 3. Results and Discussion

### 3.1. Hydrogenation of C25

#### 3.1.1. Dynamics

**Figure 2:**
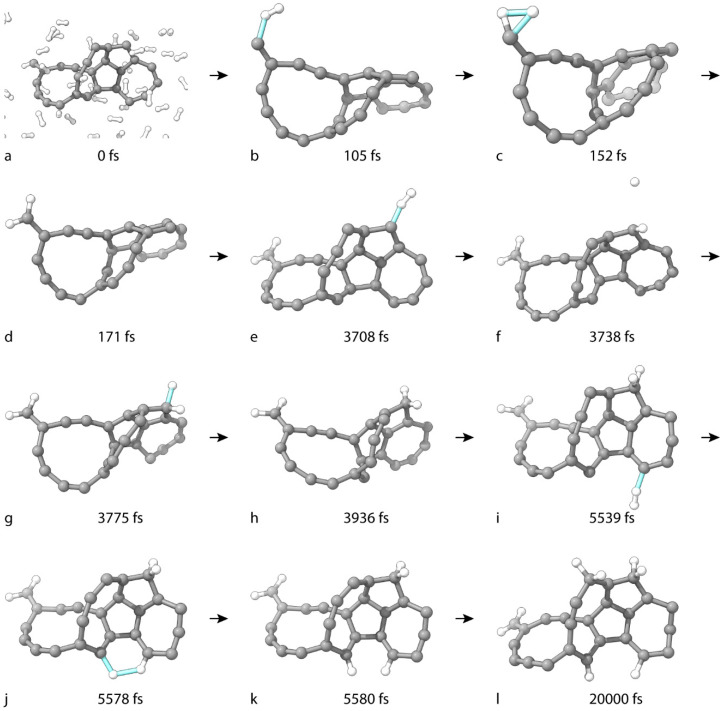
Geometries of critical structures in the formation of C_25_H_8_ from C_25_. (**a**) is the zeroth frame of the simulation. (**b**,**c**,**e**,**g**,**i**,**j**) are TS structures. (**d**,**f**,**h**,**k**) are intermediate molecules (**l**) is the final geometry of C_25_H_8_. For clarity, H_2_ molecules with no covalent interactions toward the C_25_ moiety throughout the simulation are not displayed.

The first reactive simulation of C_25_ involves 58 H_2_ molecules in an orthorhombic PBC cell with dimensions 11 Å × 12 Å × 17 Å. We considered simulations within a timeframe of 50 ps, which is sufficient for the chemical process to occur. Figure 2a illustrates the system in the initial frame of the dynamics. The final product has the net formula C_25_H_8_. All critical geometries in the simulation processes are shown in Figure 2. The TS of the first H addition occurs at 105 fs (Figure 2b). The remaining H atom binds to the same C atom, with a TS at 152 fs (Figure 2c). The remaining H–H bond dissolves simultaneously with the formation of the second C–H bond. The product is C_25_H_2_ (Figure 2d). This mechanism, like most of those in this study, involves a long-range covalent interplay between the atoms. As long as the remaining atom from the H_2_ molecule does not translate freely through the supercell, the non-diminishing forces of C–H attraction remain. Situations involving the gas-phase vacation of a free H^•^ radical are explicitly mentioned in the text. The involved carbon atom adopts sp^3^ hybridization. The TS structure of the third H-atom addition is at 3708 fs (Figure 2e). The product is shown in Figure 2f. Again, there is a long-range covalent interplay with both atoms of the gas molecule and the same carbon, but at larger distances (Figure 2f vs. Figure 2c). The TS of the addition of the remaining H atom occurs at 3775 fs (Figure 2g). The product is C_25_H_4_ (Figure 2h). The reactive C atom assumes sp^3^ hybridization. The fifth addition occurs at 5539 fs (TS in Figure 2i). This time, the remaining H atom does not completely detach until it engages in an addition TS to another C atom at 5578 fs (Figure 2j). The product is C_25_H_6_ (Figure 2k). The two engaged C atoms transition to sp^2^ hybridization. The final product of the dynamics has the net formula C_25_H_8_. Two H atoms are now bonded to C atoms 4, 15, and 19.

A single H atom is bonded to C atoms 6 and 18. It is not possible to visualize any TSs because the participating H_2_ molecule originates from a distant equivalent PBC box in the infinite supercell. However, an atom wrap procedure yields the product geometry (Figure 2l). The participating C atom assumes sp^3^ hybridization. According to the six traceable reactions out of the eight reactions, H_2_ always undergoes addition with both atoms and never leaves an unstable H^•^ radical in the system.

**Figure 3:**
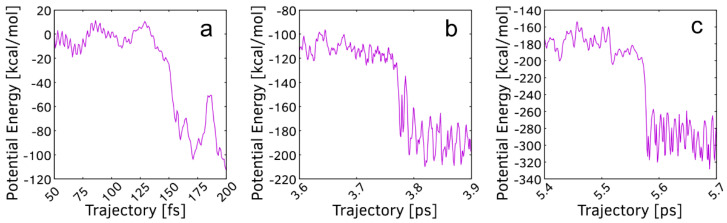
Potential energy profiles for the formation of C_25_H_8_ from C_25_. Continuous abscissa and ordinate of one simulation are split between the three diagrams. The abscissa represents continuous moments of time during the simulation. The ordinate (potential energy) is zeroed at 50 fs of the simulation. (**a**) potential energy profile of the first H_2_ addition; (**b**) potential energy profile of the second H_2_ addition; (**c**) potential energy profile of the third H_2_ addition.

The potential energy change during the addition of the first atom of a H_2_ molecule always occurs within fluctuations in the energy profile, while the addition of the second atom is always associated with a sharp plummet (Figure 3). The energy stabilization for the first, second, and third additions of the remaining H atom of each H_2_ molecule (Figure 3a–c) are 49, 61, and 116 kcal/mol, respectively, if we do not account for the large fluctuations after the reaction. When accounting for fluctuations, the energy stabilizations yield 49, 38, and 90 kcal/mol, respectively. C_25_ is expected to be a highly reactive cluster since consecutive hydrogenations appear barrierless and result in significant stabilization.

#### 3.1.2. Metadynamics

**Figure 4:**
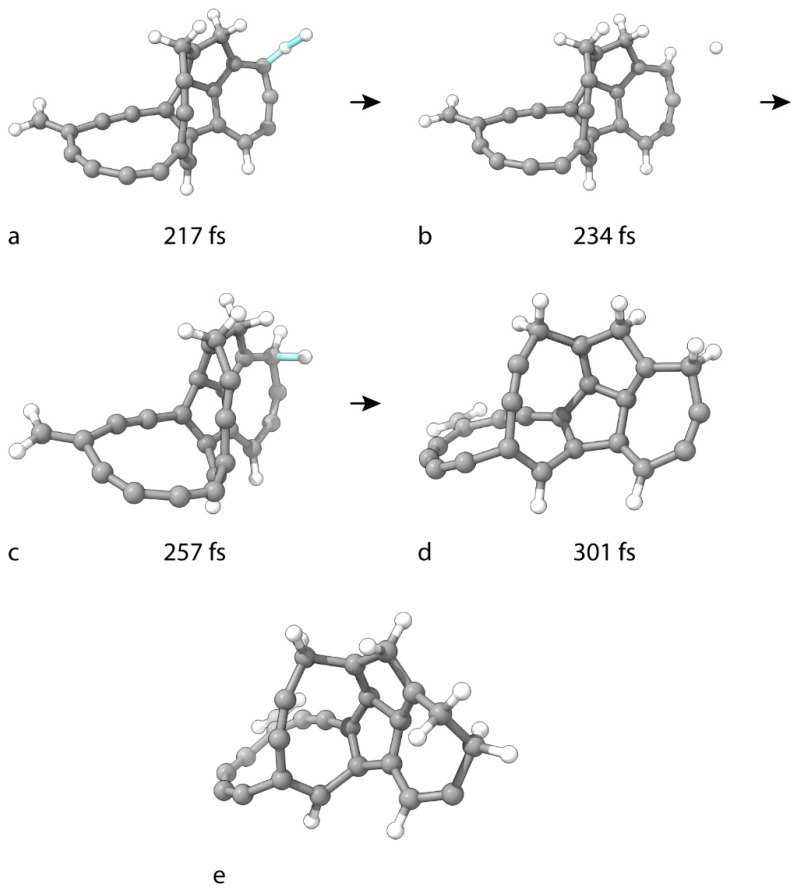
Geometries of critical structures in the subsequent hydrogenation of C_25_H_8_ to (**d**) C_25_H_10_ and (**e**) C_25_H_12_. (**a**,**c**) are TS structures. (**b**) is an intermediate molecule. The cluster geometry in the first frame in the formation of (**d**) is shown in Figure 2l.

The final product of the BOMD hydrogenation of C_25_ is C_25_H_8_. Its structure has an overall low degree of saturation. Metadynamics is used for further reactions of hydrogen addition. In all simulations, the cell dimensions are 13 Å × 13 Å × 13 Å, and only one CV is used, namely the distance between the target C atom and an H atom of the attacking H_2_ molecule. The TS in the first simulation is reached barrierlessly after 217 fs (Figure 4a). At this saddle point, the C–H distance of 1.51 Å corresponds to a partial bond. Interestingly, however, the H–H distance retains a value corresponding to a product type of bond. A product type C–H bond length of 1.07 Å is achieved at 234 fs (Figure 4b). Upon reaching the product geometry, the distance between the reactive C(5) atom and the remaining H atom (3.29 Å) appears too large for even weak covalent interactions. However, the calculation shows that this hydrogen does not leave the vicinity to travel freely through the supercell. Instead, the particle returns to attack the same carbon, and the two atoms participate in an addition TS at 257 fs (Figure 4c). This reaction step is also barrierless. At the saddle-point geometry, the C–H distance is 1.46 Å. The resulting product (C_25_H_10_) is shown in Figure 4d. The reactive C(5) atom adopts sp^3^ hybridization.

The simulation setup for the hydrogenation of C_25_H_8_ is repeated for C_25_H_10_. The product C_25_H_12_ (Figure 4d) forms similarly to its predecessor. During the process, the engaged C(25) atom adopts sp^3^ hybridization. The reactions are barrierless.

**Figure 5:**
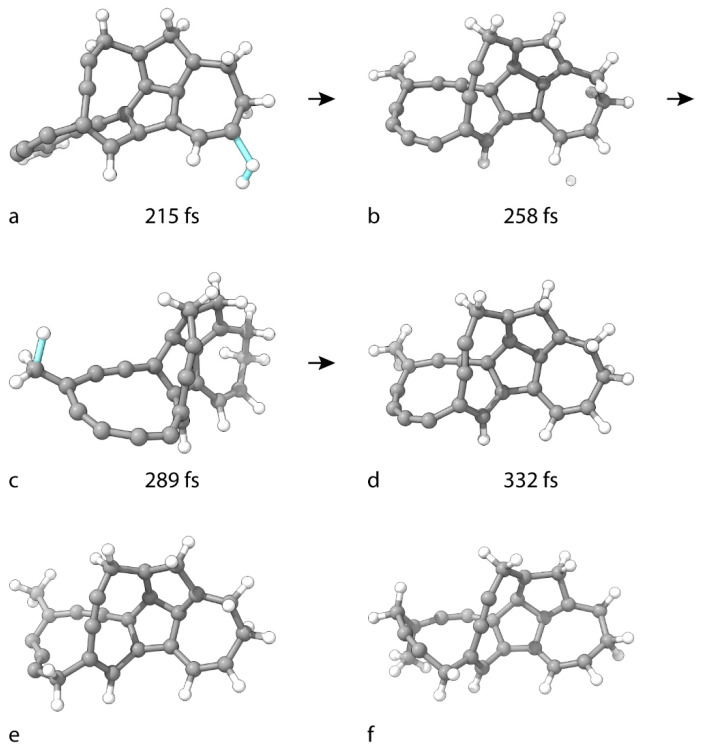
Geometries of critical structures in the consecutive hydrogenation of C_25_H_12_ to (**d**) C_25_H_14_, (**e**) C_25_H_16_, and (**f**) C_25_H_18_. (**a**,**c**) are the TS structures for both steps of the hydrogenation of C_25_H_12_ to C_25_H_14_. (**b**) is the geometry of the C_25_H_13_ intermediate. The cluster geometry in the first frame in the formation of (**d**) is shown in Figure 4e.

The simulation setup is repeated for the hydrogenation of C_25_H_12_. The TS of the first H-atom addition is reached barrierlessly at 215 fs (Figure 5a). In the completely unsaturated cluster, the reactive C(12) atom has a similar topological position as the previously participating C(25) and C(5) atoms. The geometry of the product C_25_H_13_ is shown in Figure 5b. After the reaction, the C(12) atom assumes sp^2^ hybridization. Immediately after the reaction, the remaining atom of the attacking H_2_ appears to be at a distance beyond the vanishing covalent interactions, quickly traveling through several PBC boxes of the supercell and finally binding to C(15). This now tetravalent carbon atom adopts sp^3^ hybridization. The TS is reached barrierlessly at 289 fs (Figure 5c). The geometry of the resulting product, C_25_H_14_, is shown in Figure 5d.

The hydrogenation of C_25_H_14_ to C_25_H_16_ (Figure 5e) is similar to that for C_25_H_8_ to C_25_H_10_. Both H atoms of the attacking H_2_ molecule are attached to C(17). In the product geometry, this C atom assumes sp^3^ hybridization. C_25_H_18_ is obtained in a similar way (Figure 5f), and the reactive C(21) atom also adopts sp^3^ hybridization.

**Figure 6:**
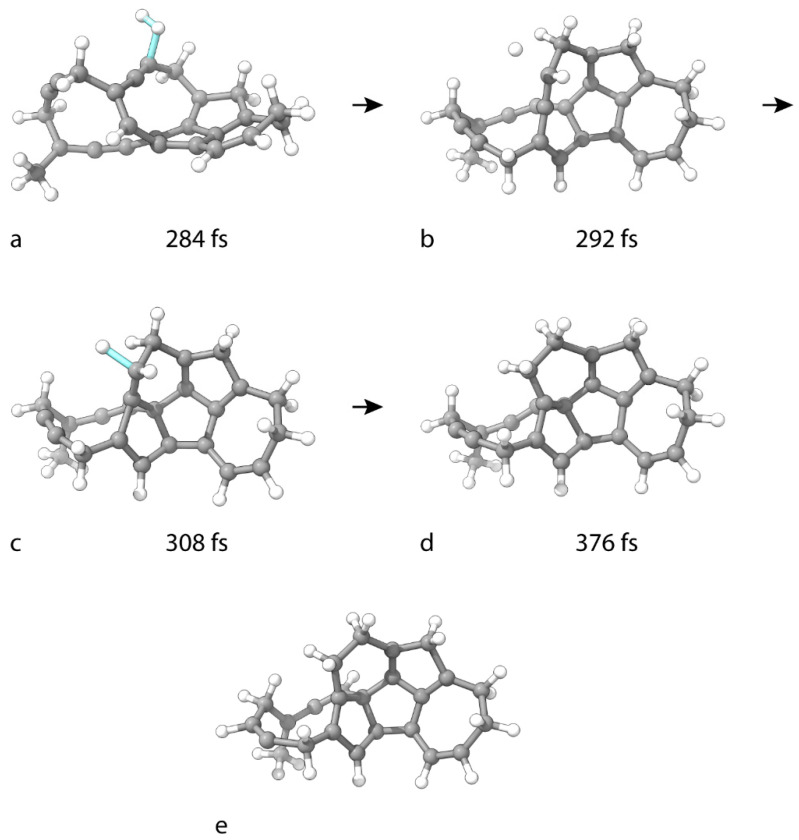
Geometries of critical structures in the consecutive hydrogenation of C_25_H_18_ to (**d**) C_25_H_20_ and (**e**) C_25_H_22_. (**a**,**c**) are TS structures for both hydrogenation steps of C_25_H_18_ to C_25_H_20_. (**b**) is the geometry of the C_25_H_19_ intermediate. The cluster geometry in the first frame in the formation of (**d**) is given in Figure 5f.

An unbiased intramolecular cyclization through a bond formation between the spatially adjacent C(1) and C(9) atoms occurs during the hydrogenation of C_25_H_18_ to C_25_H_20_. The process begins with a familiar type of TS for the first hydrogen addition, which occurs at 284 fs (Figure 6a).

Essentially, the mechanism of C_25_H_8_ saturation repeats. The second H addition occurs simultaneously with the cyclization. The TS structure is located at 308 fs (Figure 6c). The geometry of the final product, C_25_H_20_, is shown in Figure 6d. During the reaction, C(1) adopts sp^2^ hybridization, while C(9) assumes sp^3^ hybridization. The C atom participating in hydrogenation, namely, C(10), also assumes an sp^3^ state. The process takes place without barriers.

The hydrogenation of C_25_H_20_ to C_25_H_22_ (Figure 6e) is similar to that of C_25_H_12_ to C_25_H_14_. The first H atom of the attacking H_2_ molecules binds to C(11). After traveling through the supercell, the remaining H atom binds to C(16). In C_25_H_22_, carbon atoms C(11) and C(16) are in the sp^2^ hybridization state. The processes occur without barriers.

**Figure 7:**
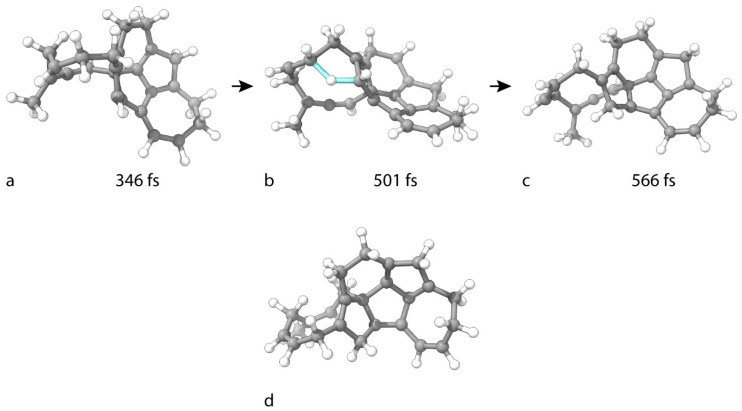
Geometries of critical structures in the consecutive hydrogenation of C_25_H_22_ to (**c**) C_25_H_24_ and (**d**) C_25_H_26_. (**a**) is an intermediate before an intramolecular H transfer. (**b**) is the TS of the H transfer. The cluster geometry in the first frame in the formation of (**c**) is given in Figure 6e.

C_25_H_24_ forms similarly to C_25_H_10_. Both H atoms bind to C(23), which changes its hybridization to sp^3^. From topological considerations, the product is a radical, as the reaction leaves the neighboring C(16) atom with a single electron in a lonely p-orbital. The original structure, with net formula C_25_H_24_ (Figure 7c), is an intermediate, and after 230 fs, a new TS is reached without applied bias, in which one of the H atoms participating in the hydrogenation simultaneously leaves the target C(23) atom and reattaches to C(18) (Figure 7b). The migration process stabilizes the cluster, as C(23) changes its hybridization to sp^2^ and forms a π bond with the unpaired electron at C(16). The geometry of the final C_25_H_24_ isomer is shown in Figure 7c. The product is no longer a radical. The reaction steps are barrierless.

The hydrogenation of C_25_H_24_ to C_25_H_26_ is akin to that of C_25_H_12_ to C_25_H_14_. The first atom of the attacking H2 binds to the CV-targeted C(24), which adopts sp^2^ hybridization and forms a π bond with C(3). After traveling through the supercell, the remaining H atom binds to C(11), which enters an sp^3^ hybridization state. The optimized geometry of C_25_H_26_ is shown in Figure 7d.

Further attempts to saturate the cluster with hydrogens are not barrierless, as C_25_H_26_ is a stable molecule. Three isolated double bonds exist between the following C-atom pairs: 1 and 2; 3 and 24; and 16 and 23. An 8-electron conjugated π system is formed by atoms 6, 7, 8, 12, 13, 14, 20, and 22.

### 3.2. Amination of C_25_

#### 3.2.1. Dynamics

**Figure 8:**
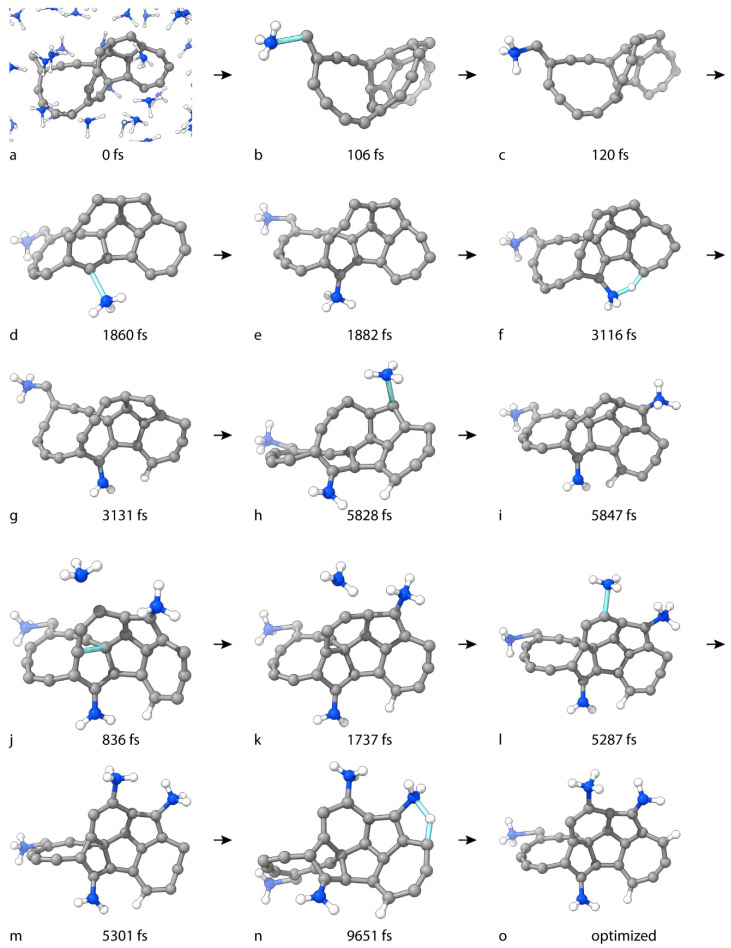
Geometries of critical structures in the formation of (**i**) C_25_H(NH_3_)_2_NH_2_ and (**o**) C_25_H_2_(NH_3_)_2_(NH_2_)_2_. (**a**) is the zeroth frame of the simulation for the initial amination of C_25_ to C_25_H(NH_3_)_2_NH_2_. (**b**,**d**,**f**,**h**) are TS structures. (**c**,**e**,**g**) are intermediate molecules. (**j**,**l**,**n**) are the TSs in the formation of (**o**). (**k**,**m**) are the intermediates in the formation of (**o**). (**i**) is the cluster geometry in the zeroth frame of the Metadynamics-modeled amination of (**i**–**o**). For clarity, NH_3_ molecules with no covalent interactions with the carbon cluster throughout the simulation are not displayed.

**Figure 9:**
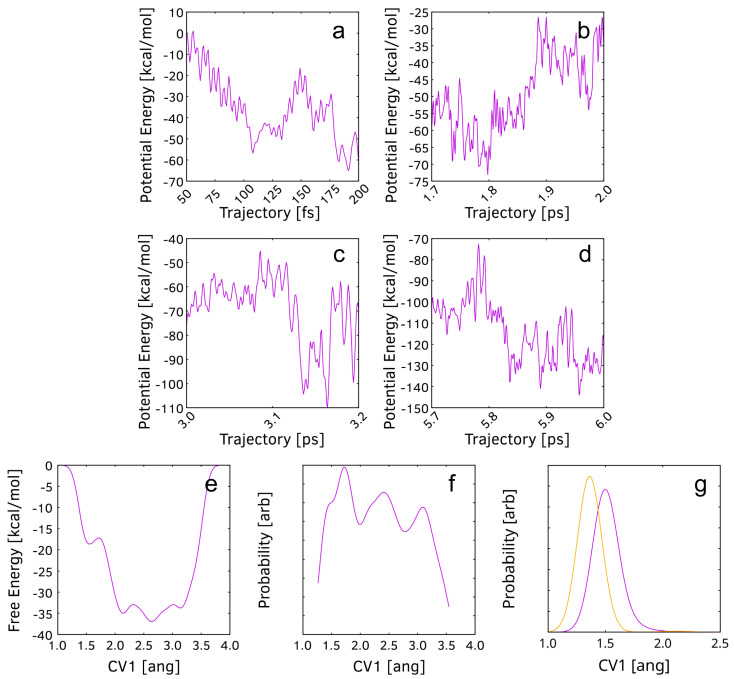
Potential energy profiles in the formation of C_25_H(NH_3_)_2_NH_2_. RDF of the C–N distance in the case of the primary amino group, bound to C(18) (yellow), and the quaternary amino group, bound to C(15) (violet). Potential energy profile of: (**a**) the ammonia addition to C(15), (**b**) the ammonia addition to C(18), (**c**) the H-shift from the second quaternary ammonium group to C(6), and (**d**) the ammonia addition to C(19). (**e**) is the FE profile of the fourth (and final) ammonia addition, resulting in C_25_H_2_(NH_3_)_2_(NH_2_)_2_. (**f**) is the RDF of the C(4)–N distance for the final addition of the amino group. (**g**) is the comparison between the RDFs of the C(18)–N and the C(15)–N distances.

The second reactive simulation of C_25_ is performed with 41 NH_3_ molecules in an orthorhombic PBC cell with dimensions 11 Å × 12 Å × 17 Å. We considered the simulations within the timeframe of 50 ps, which is sufficient for the chemical process to occur. The initial frame of the dynamics is shown in Figure 8a. After four reactions, the final product has the net formula C_25_H_9_N_3_. All critical geometries in the simulation processes are shown in Figure 8. The first reaction involves NH_3_ addition to the most unsaturated and reactive C(15) atom. The TS occurs at 106 fs (Figure 8b). The product molecule contains a quaternary ammonium group that is attached to C(15). There is a positive charge, centered at the N atom, and a negative charge, delocalized in the σ skeleton of the conjugated π system (πI) to which C(15) belongs. This process takes place without a barrier and reduces the potential energy of the system by 23 kcal/mol (Figure 9a). The second reaction is also an NH_3_ addition, leading to a new quaternary ammonium group. The TS occurs at 1860 fs (Figure 8d). The N atom is bound to C(18). The negative charge is delocalized in a larger conjugated π system (πII) than the previous one. No barrier or drop in potential energy is observed for this process (Figure 9b). The product of this elementary step is only an intermediate. The following reaction is unbiased: the H atom from the positively charged -N(+)H_3_ fragment is abstracted by C(6). The TS occurs at 3116 fs (Figure 8f). This reaction is barrierless and exothermic by 41 kcal/mol (Figure 9c). It is expected that the mobile H species is a cation, which relocates the positive charge of the ammonium group to πII. This spontaneous reaction leads to system stabilization for four reasons: (i) the absence of a positive charge on the preferably electronegative N atom, (ii) the cancellation of two opposite charges, (iii) the reduction in the absolute value of the total charge of two substituents, and (iv) the conjugation between the newly formed amino group and the carbon skeleton. Both the primary and the quaternary amino groups are bound to the rest of the carbon moiety by σ bonds, yet the oscillations of the bond length of the former have lower values, indicating higher stability (Figure 9g). The fourth and final reaction is an NH_3_ addition to C(19). The process is similar to the first ammonia addition. The TS occurs at 5828 fs (Figure 8h). The product has the net formula C_25_H_9_N_3_ (Figure 8i). The negative charge is distributed in the πII system. The chemical changes decrease the system’s potential energy by 27 kcal/mol (Figure 9d).

The lack of noticeable stabilization associated with the addition of the second ammonia is attributed to thermal fluctuations, considering the apparent stabilization effects of the first and third additions. A similarity between the second and the third addition increases the probability for this explanation: the reactions attach equivalent substituents to the πII system.

The increasingly negative charge induced in the carbon skeleton of the cluster, due to reactions resulting in quaternary ammonium groups, has an inhibiting effect on further nucleophilic additions. Thus, the cluster’s affinity for successive ammonia additions will gradually decrease. Hence, it is expected that future reactions will no longer be barrierless.

Due to their high activation energy, ammonia additions to an alkene/alkyne fragment are rather challenging and, if attempted, they usually require special conditions and/or the use of a metal catalyst [59]. Therefore, the occurrence of three consecutive barrierless ammonia additions to C_25_ demonstrates a rather extreme reactivity of the cluster. Such a high degree of instability is expected to manifest throughout the chemistry of similar structures. Exposure to air under standard conditions would probably result in self-combustion. It is not surprising that such molecules readily assemble into higher-order structures [40]. The typical mechanisms of amination involve metal–amido or metal–imido species, but—to no surprise—the mechanisms demonstrated in this study are direct. The instability of the primal carbon cluster is a reference to its overall exotic geometry. A reasonable explanation for the high activity of C_25_ is the partial radical character of the edge atoms. The combination of bonding angles and topology results in an improper orbital overlap. The distance between the outer lobes of the p-orbitals participating in π bonds is too big, while the inner lobes are too close. Such geometry causes an increase in the weight of the diradical configuration in alkene and alkyne fragments. The stabilization due to π bonding remains suboptimal.

#### 3.2.2. Metadynamics

Metadynamics is employed for the additional amination of the final product of regular, unbiased dynamics. The dimensions of the orthorhombic PBC cell are 13 Å × 13 Å × 13 Å. Only one CV is used, namely the C–N distance. The first reaction to occur is an unbiased, intramolecular cyclization with a bond between C(1) and C(9). The TS is at 836 fs (Figure 8j). The product geometry is shown in Figure 8j. Once formed, the bond remains throughout the rest of the simulation. The covalent C–N distance of a product type appears in the amination at C(4) via the TS structure found at 5287 fs (Figure 8l). The formula of the product is C_25_H_2_(NH_3_)_3_NH_2_ (Figure 8m). The forward and reverse free-energy barriers are 20 kcal/mol and 1.5 kcal/mol, respectively (Figure 9e). For reasons mentioned earlier, the cluster has exceeded its capacity for barrierless amination. The radial distribution function (RDF) of the C–N distance is shown in Figure 9f. The endothermicity of the forward reaction causes the reagents to be much more dominant along the trajectory. The cluster with a peak at 1.73 Å signifies an intermolecular attraction. The inflection point at 1.49 Å separates the left-most cluster, representing C–N bond length values of the product type.

The rise in energy leads to an additional and unbiased reaction. A H atom from the quaternary ammonium group at C(19) is abstracted by the neighboring C(5) atom. The TS structure is at 9651 fs (Figure 8n). The product is labeled as C_25_H_2_(NH_3_)_2_(NH_2_)_2_ (Figure 8o). This quadruply aminated final product includes two systems of conjugated π-electronic density. One of them is almost planar and consists of eight electrons. The other system spans most of the molecule, includes 20 electrons, and involves cyclic out-of-plane density over condensed rings.

## 4. Conclusions

We modeled the processes of hydrogenation and amination of a primal carbon cluster of the tangled polycyclic type in space. The reagents for the addition reactions were H_2_ and NH_3_, respectively. The cluster is the product of the spontaneous stabilization of a non-covalent aggregate of 25 carbon atoms with randomized positions. DFTB2 Born–Oppenheimer Molecular Dynamics was employed to model the initial chemical processes, and DFTB2 Metadynamics was utilized to accelerate the saturation. All transition states were identified, revealing multiple mechanisms for each type of reaction. The reasons behind all the chemical events and characteristics of the critical systems are given in terms of electronic effects. An unbiased reaction of intramolecular cyclization occurs during both hydrogenation and amination. The final product of barrierless hydrogen saturation is C_25_H_26_. In this molecule, there are three isolated double bonds and an 8-electron π-delocalized system, with no triple bonds present in the final structure. The addition of ammonia always begins with the formation of a quaternary ammonium group. Although such reactions are auto-inhibitory, the process occurs three times before the emergence of an energy barrier. In comparison with typical alkenes, alkynes, and annulenes, primal carbon clusters appear to be highly activated. The mechanism of the amination of C_25_ is unlike those in organic chemistry. The process does not require catalysis and does not involve intermediates. In recurring amination, a barrier arises more quickly than in repetitive hydrogenation. A proton abstraction from a –N(+)H_3_ group by a neighboring carbon atom occurs twice as a form of unbiased stabilization. The product of the final guided and highly exothermic NH_3_ addition is the unstable C_25_H_2_(NH_3_)_2_(NH_2_)_2_. This final product includes two conjugated π systems. One of them is almost planar and involves eight electrons. The other system consists of 20 electrons delocalized over multiple condensed rings. It is characterized by cyclic out-of-plane density.

## Data Availability

Data are contained within the article and Supplementary Materials.

## Supplementary Materials

The following supporting information can be downloaded at: https://www.mdpi.com/article/10.3390/nano15141110/s1, Table S1. Parameters of the production simulations: reaction (simulation), type of calculation, SCF method, ensemble, temperature [K] and pressure.

## Abbreviations

The following abbreviations are used in this manuscript:

BOMD	Born–Oppenheimer Molecular Dynamics
CSVR	Canonical Sampling through Velocity Rescaling
CV	Collective Variable, Colvar
DFT	Density Functional Theory
DFTB	Density Functional Theory Tight Binding
FE	Free Energy
KS	Kohn–Sham
MTD	Metadynamics
PBC	Periodic Boundary Conditions
RDF	Radial Distribution Function
SCC	Self-Consistent Charges
SCF	Self-Consistent Field
TS	Transition State
