# Supplementary material for: An Ab Initio Metadynamics Study Reveals Multiple Mechanisms of Reactivity by a Primal Carbon Cluster Toward Hydrogen and Ammonia in Space
Source: Nanomaterials (Basel). 2025 Jul 17;15(14):1110. doi: 10.3390/nano15141110 (PMC12300080; doi:10.3390/nano15141110)
Supplement: Supplementary file 1 [file nanomaterials-15-01110-s001.zip › nanomaterials-3715806-supplementary.pdf]

Ab initio metadynamics study revealing multiple mechanisms of reactivity of a primal carbon cluster towards hydrogen and ammonia in space

SUPPORTING INFORMATION

Dobromir A. Kalchevski[1], Stefan K. Kolev[1], Dimitar, V. Trifonov[1], Ivan G. Grozev[1], Hristiyan A. Aleksandrov[2], Valentin N. Popov[3], Teodor I. Milenov[1]

[1] "E. Djakov" Institute of Electronics- Bulgarian Academy of Sciences, 72 Tzarigradsko Chausee Blvd., 1784 Sofia, Bulgaria
[2] Faculty of Chemistry and Pharmacy, Sofia University "St. Kliment Ohridski", 1 J. Bourchier Blvd., Sofia 1164, Bulgaria
[3] Faculty of Physics, Sofia University "St. Kliment Ohridski", 5 J. Bourchier Blvd., Sofia 1164, Bulgaria

Table S1. Parameters of the production simulations: reaction (simulation), type of calculation, SCF method, ensemble, temperature [K] and pressure.

simulation	type	SCF method	ensemble	temperature	pressure
		hydrogenation			
$C_{25} \rightarrow C_{25}H_8$	BOMD[a]	DFTB2	NVT	400	variable
$C_{25}H_8 \rightarrow C_{25}H_{10}$	MTD[b]	DFTB2	NVT	400	variable
$C_{25}H_{10} \rightarrow C_{25}H_{12}$	MTD	DFTB2	NVT	400	variable
$C_{25}H_{12} \rightarrow C_{25}H_{14}$	MTD	DFTB2	NVT	400	variable
$C_{25}H_{14} \rightarrow C_{25}H_{16}$	MTD	DFTB2	NVT	400	variable
$C_{25}H_{16} \rightarrow C_{25}H_{18}$	MTD	DFTB2	NVT	400	variable
$C_{25}H_{18} \rightarrow C_{25}H_{20}$	MTD	DFTB2	NVT	400	variable
$C_{25}H_{20} \rightarrow C_{25}H_{22}$	MTD	DFTB2	NVT	400	variable
$C_{25}H_{22} \rightarrow C_{25}H_{24}$	MTD	DFTB2	NVT	400	variable
$C_{25}H_{24} \rightarrow C_{25}H_{26}$	MTD	DFTB2	NVT	400	variable
		amination			
$C_{25} \rightarrow C_{25}H_9N_3$	BOMD	DFTB2	NVT	400	variable
$C_{25}H_9N_3 \rightarrow C_{25}H_{12}N_4$	MTD	DFTB2	NVT	400	variable

[a] Born-Oppenheimer Molecular Dynamics.

[b] Metadynamics.
